# A Multiomics Framework to Unlock the Relationships between Wine, Food, and Gut Health

**DOI:** 10.1016/j.advnut.2025.100468

**Published:** 2025-06-21

**Authors:** Antoine Abrieux, Mariana Barboza, Kristin Hirahatake, Hunter Jacobs, Harold H Schmitz, Sean H Adams, Justin B Siegel

**Affiliations:** 1 Innovation Institute for Food and Health, University of California Davis, Davis, CA, United States; 2 Genome Center, University of California Davis, Davis, CA, United States; 3 Graduate School of Management, University of California, Davis, CA, United States; 4 Department of Surgery, University of California Davis School of Medicine, Sacramento, CA, United States; 5 Center for Alimentary and Metabolic Science, University of California Davis School of Medicine, Sacramento, CA, United States; 6 Department of Chemistry, University of California Davis, Davis, CA, United States; 7 Department of Biochemistry and Molecular Medicine, University of California Davis, Davis, CA, United States

**Keywords:** metabolomics, microbiome, microbiota, food matrix, alcohol

## Abstract

Wine has long been studied for its cardioprotective effects, exemplified by the French paradox—the observation of relatively lower cardiovascular disease (CVD) rates in the French population despite high dietary cholesterol and saturated fat intake, historically attributed to resveratrol and other bioactive factors from wine consumption. Recent findings suggest that the moderate consumption of wine could impact health well beyond CVD risk, including effects on intestinal physiology and gut microbial diversity and function. For example, wine contains a rich array of polyphenols, organic acids, and oligosaccharides, which may interact with the gut microbiota to alter microbial communities and to promote metabolism of wine-derived compounds into a diverse range of xenometabolites (microbe-produced metabolites) with local and systemic effects on the host. This interplay underscores the potential mechanisms by which moderate wine consumption impacts gut and systemic health. Furthermore, because wine is often consumed with meals, there is a critical need to understand how specific foods intersect with wine’s chemical complexity to influence physiology in the gut and body-wide. This review explores how advancements in multiomics technologies can be leveraged to characterize wine’s “dark matter” and to consider interactions of wine components with complex food matrices to influence gut health. This framework holds potential to enhance our understanding of how moderate consumption of wine influences health and to inform the development of functional food innovations derived from wine’s molecular components.

**Statement of significance**

This review explores current evidence regarding the impact of moderate wine consumption on human health, with a focus on its interactions with gut physiology and metabolism. It provides an innovative framework, leveraging advancements in multiomics to enable research to decipher the understudied interplay between wine’s bioactive compounds, food matrices, gut functions, and health.

## Introduction

Wine is one of the most ancient and celebrated beverages in human history, enjoyed across cultures for its flavor, complexity, and social significance. The relationship between wine consumption and human health has captured scientific interest for decades. Because the first observations of the French paradox—low cardiovascular disease (CVD) rates in France despite a diet high in saturated fats and cholesterol, and regular wine consumption—were published in the early 1990s, researchers have sought to understand the mechanism(s) behind this phenomenon [1]. A recent United States Surgeon General’s advisory report highlights the association between regular alcohol consumption and increased cancer risk [2]. However, the context of intake and compositional differences between sources of alcohol were not considered. A review of evidence on alcohol and health from the National Academy of Sciences [3] and multiple articles in the extant literature [[4], [5], [6], [7], [8], [9], [10], [11], [12], [13], [14]] provide a more nuanced perspective to alcohol intake and health: the association between moderate consumption of wine—defined as 2 drinks/d for males and 1 drink/d for females per the 2020 Dietary Guidelines for Americans—is associated with a lower risk of all-cause mortality.

**FIGURE 1:**
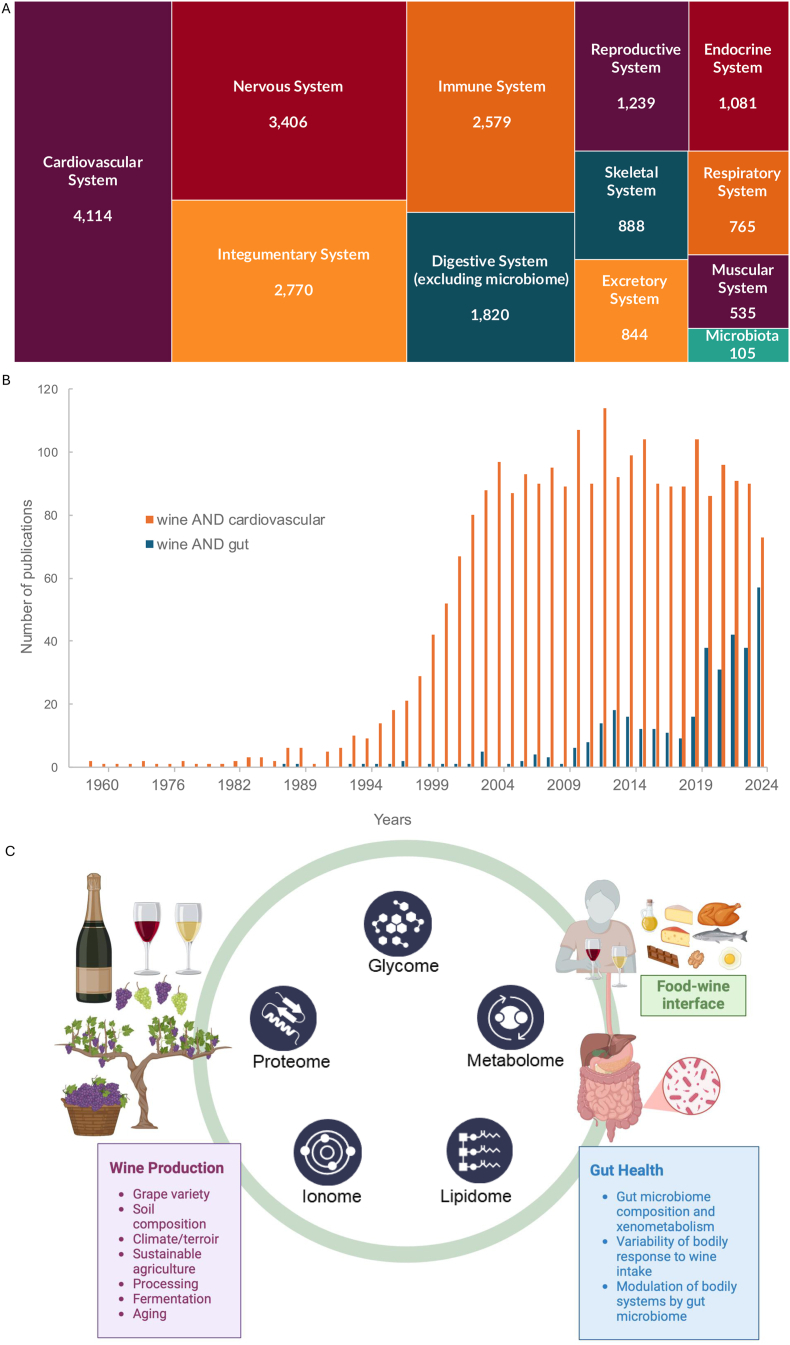
There is a growing interest in wine and health research across the major physiological systems, including the gastrointestinal tract. (A) Tree map highlighting publication numbers for the search terms “wine AND” [the major physiological system], including the gastrointestinal tract with 105 articles related to microbiota (refer to Supplemental Table 1). (B) An illustration of a trend in publications reporting on wine and the gut compared with wine and the cardiovascular system. Data shown are the number of research articles on PubMed using the search terms “wine AND cardiovascular” and “wine AND gut” between 1957 and 2025. (C) A proposed multiomics framework to decipher molecularly defined 3-way relationships among wine, food, and gut health.

To date, efforts to elucidate the mechanisms responsible for the beneficial effect of moderate wine consumption on the cardiovascular system have often focused on resveratrol, a polyphenol found predominantly in red wine that has been extensively studied for its antioxidant, anti-inflammatory, and cardioprotective properties [[15], [16], [17], [18]]. Red and white wines, however, contain numerous polyphenols, which are a complex mixture of flavonoids (for example, anthocyanins and flavan-3-ols) and nonflavonoids (for example, cinnamates, vescaligin, caffeic and gallic acid) [19]. Notably, flavan-3-ols, a class of flavonoids abundant in both wine and cocoa, have been identified as key dietary bioactives associated with improved cardiometabolic health, as recognized in recent dietary guidelines for flavan-3-ol intake [20]. Compared with white wine, red wine contains ∼10-fold more phenolic compounds as a result of the fermentation of grape juice with skins, grape pieces, and seeds [21]. Active compounds such as tyrosol, caffeic acid, and shikimic acids have been identified in white wine and may contribute to its cardioprotective properties [14,[22], [23], [24], [25]]. Beyond CVD, there is increasing attention to the impact of wine consumption on health across all of the main physiological systems (Figure 1A; Supplemental Table 1), including a growing body of research focused on wine and the gut (Figure 1B).

These findings highlight the necessity of shifting the research paradigm toward a more holistic view of wine consumption and its implications on health beyond CVD, and considering potential mechanisms by which complex wine components interact with the body. The rise of advanced omics technologies—including metabolomics, proteomics, lipidomics, and glycomics—has revolutionized the ability to unmask food’s intricate chemical composition, uncovering thousands of compounds that may work singly or synergistically to influence health outcomes [26]. These technologies, and advances in data science, are opening up the opportunity to evaluate the potential health impacts of a given dietary component (for example, cholesterol, sugars, saturated fats) in the context of a diet pattern and a molecularly defined food matrix, as opposed to considering the impact of an isolated compound [[27], [28], [29]]. This narrative review considers observational studies from human clinical trials, cell model experiments, and “omics” analyses from the past 15 y to highlight how multiomics approaches can help to address current knowledge gaps regarding the potential roles and interactions of wine and food matrices in human physiology, with an emphasis on gut health (Figure 1C).

## Wine and Gut

The gastrointestinal tract is the first point of contact for wine, whether consumed alone or with food. Hence, this organ system and the microbial communities that inhabit the gut are logical sites of action for wine-associated compounds and interactions with food- and microbe-derived factors. As an illustration, polyphenols undergo extensive transformation before entering the bloodstream. It is estimated that 5%–10% of ingested polyphenols are absorbed in the small intestine, whereas the remaining 90%–95% reach the colon where they undergo metabolism by the microbiota into a variety of bioactive phenolic metabolites, lactones, and phenolic acids, a fraction of which are subsequently absorbed to reach the systemic circulation [30,31].

The functions of the gut microbiota are increasingly recognized as a cornerstone of human health and well-being, influencing a multitude of physiological and health-related processes, including nutrient absorption, energy homeostasis, cardiovascular and immune function, and cognition [[32], [33], [34], [35], [36]]. The diverse microbial community inhabiting the gut interacts dynamically with dietary components, breaking down complex molecules and converting nondigestible food compounds (including resistant starches and fibers) and phytochemicals such as polyphenols into secondary metabolites that harbor a wide range of bioactive and protective properties [37]. This intricate relationship between ingested metabolites and the gut microbiota is central to understanding how specific dietary patterns, foods, and moderate wine consumption can shape health outcomes at both local and systemic levels.

Over the last 10–15 y, evidence of the potentially beneficial effect of wine consumption on gut health has emerged, with several small studies showing an effect of moderate red wine consumption on the gut microbial diversity in humans [[38], [39], [40]]. In their systematic review, Nash et al. [39] identified 7 studies looking at the effect of grape and wine polyphenols on the gut microbiota in humans, with only 1 reporting changes in gut microbial communities [41] and the remaining 6 referring to gut microbiota as central to the formation of phenolic metabolites. Two randomized controlled studies in 41 healthy individuals (22 females and 19 males, age range 20–65 y) revealed changes in the microbial-derived phenolic metabolic profile of feces after moderate consumption of red wine (250 mL of red wine/d for 4 wk) [42,43]. Following up on these results, the multiomics analysis of fecal samples of 19 individuals classified as low, medium, and high wine-polyphenol metabolizers identified interindividual variability of the effect of wine consumption on gut bacterial ⍺-diversity, but more homogeneity of β-diversity among moderate wine consumers, independently of their fecal metabotype (high, medium, and low capacity to metabolize wine polyphenol) [44]. In a randomized, crossover, controlled intervention study by Queipo-Ortuño et al. [41], daily red wine consumption (272 mL/d) for 4 wk in 10 healthy male volunteers was found to reduce *Clostridium* genera abundance and potentiate the growth of “health-promoting” bacteria such as *Enterococcus, Prevotella, Bacteroides,* and *Bifidobacterium*. Specific bacterial changes with red wine were associated with reductions in lipid markers, blood pressure, and inflammation indices. The results of moderate red wine consumption on colonic microbiota of 15 healthy volunteers demonstrated an association between the consumption of 250 mL of red wine/d over 1 mo and an increase in fecal microbial diversity [45]. A crossover study by Clemente-Postigo et al. [46] revealed a significant increase in *Bifidobacteria* and *Prevotella* with red wine consumption (272 mL/d) over 3 consecutive periods of 20 d, which negatively correlated with blood LPS concentrations. Furthermore, in a randomized, crossover, controlled intervention study of 10 individuals with metabolic syndrome and 10 healthy volunteers, red wine consumption (272 mL/d) over 30 d significantly increased the number of fecal *Bifidobacteria* and *Lactobacillus* and butyrate-producing bacteria (*Faecalibacterium prausnitzii* and *Roseburia*) and decreased LPS-producing bacteria (*Escherichia coli* and *Enterobacter cloacae*). The authors concluded that these modifications in gut microbiota might be responsible for the observed improvements in metabolic syndrome markers, including reduction in blood pressure, blood glucose levels, and plasma triglyceride and total cholesterol levels [47].

A study by Haas et al. [48] investigated the effects of 3 wk of red wine consumption (250 mL/d, 5 d/wk) on the gut microbiota, plasma trimethylamine N-oxide, and the plasma metabolome in 42 males with coronary artery disease. The findings of this randomized crossover trial showed no difference in plasma trimethylamine N-oxide between the red wine intervention and alcohol abstention, but there was significant remodeling of the gut microbiota in wine-consuming participants, with an increase in β-diversity and predominance of *Parasutterella*, *Ruminococcaceae*, several *Bacteroides* species, and *Prevotella*. Recently, a cross-sectional study conducted on 3 independent cohorts including the TwinsUK (916 United Kingdom female individuals) as a discovery cohort, and the Flemish Gut Flora Project (*n* = 1104) and the American Gut Project (*n* = 904) cohorts as replicates, explored the effect of beer and cider, red wine, white wine, spirits, and sum of all alcohols on the α-diversity of the gut microbiota [49]. This analysis using a linear mixed-effect model adjusted for age, BMI, Healthy Eating Index, education, and family structure indicated a positive association between red wine consumption (and none with other alcohol categories) and gut microbiota α-diversity in a frequency-dependent manner with self-reported drinking frequency classified as never, rarely (less that once a month), occasionally (less than once a week but more than once a month), regularly (more than once a week but less than daily), and daily (at least once a day). In the studies above (summarized in Table 1) [[41], [42], [43], [44], [45], [46], [47], [48], [49]], despite correlations or associations of microbiota shifts with moderate wine consumption and improvements in cardiometabolic indices, cause–effect relationships remain to be confirmed. Furthermore, large-scale and long-term randomized controlled studies that integrate the precise molecular mapping of both wine (and co-consumed food composition) with clinical outcomes relevant to gut health are warranted. Nevertheless, this growing evidence base highlights the possible importance of gut microbiota in mediating the health effects of moderate wine consumption.

**TABLE 1:** Summary table of RCT and observational studies on wine consumption and gut health.

References	Study design	Sample size	Mean age ± SD	Wine type/dose	Duration	Outcomes associated with wine intake	Limitations
Queipo-Ortuño et al. 2012 [41]	Crossover RCT	10 healthy males	48 ± 2 y (range: 45–50 y)	Dealcoholized RW (272 mL/d), RW 272 mL/d	20 d	Modulation of gut microbiota: ↑*Bifidobacteria, Enterococcus, Prevotella, Bacteroides,* ↓*Clostridium* spp	Small sample size, male-only, short-term
Clemente-Postigo et al. 2013 [46]	Crossover RCT	10 healthy males	Chronic study: 48 ± 2 y (range: 45–50 y)	Dealcoholized RW (272 mL/d), RW 272 mL/d	20 d	↑*Bifidobacteria* and *Prevotella* negatively correlated with plasma LPS concentrations	Small sample size, male-only, short-term
Moreno-Indias et al. 2016 [47]	Crossover RCT	20 adults (10 with MetS [MetS], 10 healthy controls)	48 ± 2 y (range: 45–50 y)	Dealcoholized RW (272 mL/d), RW 272 mL/d	30 d	Positive effects on the composition of the gut microbiota and a reduction in the MetS risk markers in MetS subjects ↑*Bifidobacteria*, *Lactobacillus* (intestinal barrier protectors), *Faecalibacterium prausnitzii,* and *Roseburia* (butyrate-producing bacteria) ↓*Escherichia coli* and *Enterobacter cloacae* (LPS producers)	Male-only, short-term
Muñoz-González et al. 2013 [42]	RCT, targeted metabolomics	41 healthy subjects (22 females and 19 males/33 intervention and 8 control)	Range: 20–65 y	RW, 250 mL/d	4 wk	Changes in phenolic microbial metabolites in feces 35 phenolic metabolites identified, 10 compounds (mainly benzoic and 4-hydroxyvaleric acids) increased after the RW intake	Focus on metabolites only; short-term
Jiménez-Girón et al. 2015 [43]	RCT, untargeted metabolomics	41 healthy subjects (22 females and 19 males/33 intervention and 8 control) from Muñoz-González et al. (2013)	Range: 20–65 y	RW, 250 mL/d	4 wk	Changes in phenolic microbial metabolites in feces 37 metabolites regulated by RW intake (23 higher and 14 lower)	Focus on metabolites only; short-term
Belda et al. 2021 [44]	RCT	19 healthy subjects (10 females + 9 males from Muñoz-González et al. [2013])	Range: 25–57 y	RW, 250 mL/d	4 wk	*Phascolarctobacterium*, *Pelotomaculum* and *Prevotella*, positively correlated with polyphenol concentration in feces	Small sample size, short-term
Barroso et al. 2017 [45]	RCT	20 healthy adults (15 intervention and 5 control)	Not specified	RW, 250 mL/d	4 wk	↑ α-diversity in gut microbiota	Small sample size, short-term
Haas et al. 2022 [48]	Crossover RCT	42 males with coronary artery disease	46–69 y (age mean, 60 y)	RW, 250 mL/d every 5 d	3 wk	Remodeling of the gut microbiota and significant changes in the plasma metabolome	Small sample size, short-term
Roy et al. 2020 [49]	Cross-sectional observational	3 independent cohorts - Discovery: TwinsUK (916 UK female individuals) - Replicate 1: Flemish Gut Flora Project (FGFP) *n* = 1104 - Replicate 2: American Gut Project (AGP) *n* = 904	Linear mixed-effect model adjusted for age, BMI, Healthy Eating Index, education, and family structure	RW, WW drinking frequency categories: never (individuals who reported to never drink alcohol), rarely (less that once a month), occasionally (less than once a week but more than once a month), regularly (more than once a week but less than daily), and daily (at least once a day)	Not applicable	- RW associated with ↑ α-diversity in gut microbiota across all 3 cohorts in frequency-dependent manner even with rare consumption - Milder but positive association between WW and α-diversity in gut microbiota - No association with other alcohol categories (beer or spirits)	Observational, self-reported alcohol consumption captured differently in all cohorts, complete homogenization between datasets not possible

Abbreviations: MetS, metabolic syndrome; RCT, randomized controlled trial; RW, red wine; WW, white wine.

Additionally, wine-derived polyphenols have been considered for their potential role in the maintenance of intestinal homeostasis, a key determinant of gut health [50]. Through antioxidant properties, polyphenols help neutralize reactive oxygen species, thereby reducing oxidative stress within the intestinal environment. By scavenging free radicals, polyphenols protect the integrity of the intestinal epithelium, preventing oxidative stress and inflammation [50]. These antioxidant properties are further supported by evidence from a total peroxyl radical-trapping antioxidant potential of plasma in vitro experiment demonstrating that red wine polyphenols (1.75 μg/mL) can protect human plasma PUFAs from lipid peroxidation [51]. Wine polyphenols can also interfere with bacterial quorum sensing, reduce biofilm formation, and inhibit the adhesion of gram-negative pathogens, potentially lowering the risk of infections and dysbiosis [[52], [53], [54]]. Moreover, results from studies using simulated gastrointestinal digestion, colonic fermentation, and Caco-2 intestinal cell models suggest that red wine polyphenols, particularly quercetin and its gut microbe-derived metabolite 3,4-dihydroxyphenylacetic acid, may help preserve intestinal barrier integrity by reducing paracellular permeability [55].

**FIGURE 2:**
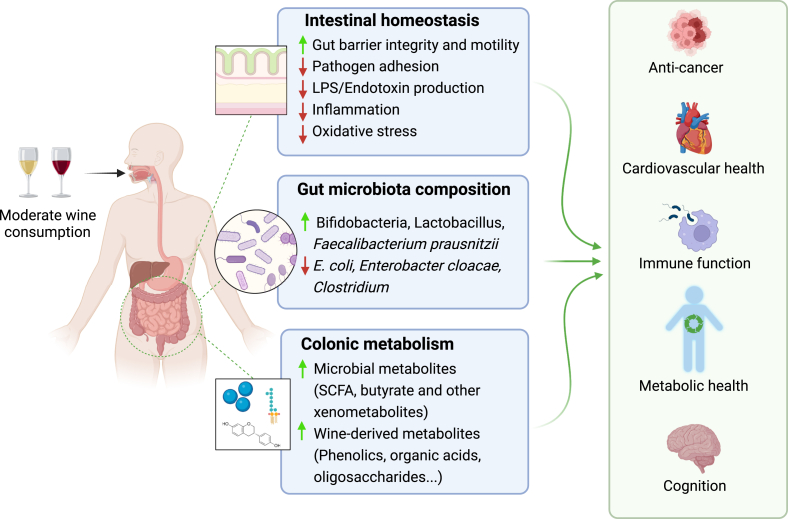
Known and potential effects of moderate wine consumption on gut and body-wide health. SCFA, short-chain fatty acid.

Although research on the effect of wine consumption and gut health has largely focused on polyphenols, wine’s complexity extends far beyond these compounds. Other bioactive components in wine include organic acids (for example, tartaric and malic acid), lipids, oligosaccharides (OS), and ethanol. Through their impact on the gut physiology and environment, these compounds can, in theory, exert effects on microbial communities and gut health [56]. For example, evidence from in vitro studies suggests that organic acids such as malic acid in white wine and succinic acid in red wine can stimulate gastric acid production, altering the upper gut pH and potentially influencing microbial populations, including *Candida* and *Lactobacilli* [[57], [58], [59]]. Additionally, the transformation of wine-associated compounds by gut microbes could result in the production of molecules such as short-chain fatty acids (SCFAs, from metabolism of wine OS) and other secondary metabolites with distinct metabolic effects [60]. Furthermore, alcohol concentrations below 15%, as typically found in wine, have been shown to accelerate gastric motility, potentially leading to improved digestion, better glycemic regulation, and reduced bloating [61] (Figure 2). Clearly, much more research is needed to fully characterize the underexplored catalog of wine components and to determine bioactivities relevant to gut health.

## Multiomics Approaches for Bioactive Discovery in Wine

The peculiar chemical matrix of wine—comprising thousands of compounds including polyphenols, organic acids, lipids, amino acids, esters, and minerals—plays a crucial role in shaping its flavor, aroma, color, texture, and potential health effects. As a product of grape fermentation by yeast, wine stands apart from most other alcoholic beverages. Unlike spirits, which undergo distillation, wine retains a diverse array of natural bioactive compounds from grapes along with microbial metabolites, OS, and peptides produced during fermentation. Recent advances in multiomics technologies have begun to unmask the “dark matter” of wine, shedding light on its components and how they may interact with the host and gut microbial systems to impact physiology (Table 2) [42,43,48,[62], [63], [64], [65], [66], [67], [68], [69], [70], [71], [72], [73], [74], [75], [76], [77], [78], [79], [80], [81], [82], [83], [84], [85], [86], [87], [88], [89], [90], [91], [92], [93], [94], [95], [96], [97], [98], [99], [100], [101], [102], [103], [104], [105], [106], [107], [108], [109], [110], [111], [112], [113], [114], [115], [116], [117], [118], [119]]. Yet, many bioactive molecules remain to be characterized and their influence on gut health is still largely unknown. Moreover, key questions persist regarding how terroir, grape variety, and vinification processes shape wine’s chemical complexity and hence its possible health implications.

**TABLE 2:** Multiomic toolbox to analyze the molecular composition of wine and associated biological properties.

“Omic” technology	Molecule type	Examples in wine	Potential biological properties	References
Metabolomics (LC/MS, GC/MS, CE-MS, NMR, FTIR)	Phenolic compounds	Resveratrol, quercetin, catechins, anthocyanins	Antioxidant, anti-inflammatory, anti-adipogenic, cardioprotective	[42,43,48,[62], [63], [64], [65], [66], [67], [68], [69], [70], [71], [72], [73], [74], [75], [76], [77]]
	Organic acids	Tartaric, malic, citric, lactic acids	Antimicrobial, metabolic regulation	
Glycomics (GC-MS, NMR, HPLC)	Carbohydrates	Oligosaccharides, polysaccharides rich in arabinose and galactose, rhamnogalacturonan	Energy source, gut microbiome modulation	[[78], [79], [80], [81], [82], [83], [84], [85], [86], [87], [88], [89], [90]]
Lipidomics (DI-MS, LC-MS, GC-MS, DESI-MS, MALDI-MS LC-MS)	Lipids	Fatty acids, phospholipids, glycolipids, sterols, sphingolipids	Membrane integrity, anti-inflammatory	[[91], [92], [93], [94], [95], [96], [97]]
Proteomics, Peptidomics (LC-MS/MS, SDS-PAGE)	Proteins/peptides	Bioactive peptides, enzymes (for example, chitinase, thaumatin-like proteins, glucosidase)	Antioxidant, ACE and PEP inhibitory activity	[[98], [99], [100], [101], [102], [103], [104], [105], [106], [107], [108], [109], [110], [111]]
Ionomics (ICP-AES, AAS), ICP-MS)	Ions	Potassium, magnesium, calcium, iron, copper	Electrolyte balance, enzymatic functions	[[112], [113], [114], [115], [116], [117], [118], [119]]

Abbreviations: AAS, atomic absorption spectroscopy; ACE, angiotensin-I-converting enzyme; CE, capillary electrophoresis; DESI, desorption electrospray ionization; DI, direct infusion; FTIR, Fourier transform infrared spectroscopy; GC, gas chromatography; ICP-AES, inductively coupled plasma atomic emission spectroscopy; LC, liquid chromatography; MALDI, matrix-assisted laser desorption/ionization; MS, mass spectrometry; NMR, nuclear magnetic resonance; PEP, prolyl endopeptidase.

### Metabolomics

Metabolomics is a powerful tool to explore the diverse chemical landscape of wine. The unprecedented resolution achieved by advanced analytical platforms such as nuclear magnetic resonance spectroscopy, mass spectrometry-based metabolomics, and chromatographic techniques continues to unveil thousands of previously uncharacterized metabolites in wine [62,63]. Currently, Fourier transform ion cyclotron resonance mass spectrometry offers the highest mass resolution, making it the most powerful technique for analyzing complex samples. It enables the identification of over 200,000 compounds in a single analysis, providing the most comprehensive characterization of the chemical fingerprint of wine although allowing rapid, high-throughput profiling without chromatographic separation [64].

The complexity of wine’s metabolite fingerprint is shaped by multiple factors, including grape variety, terroir, fermentation dynamics, and aging processes [65,66]. For instance, regional differences in soil composition, climate conditions, and vineyard management practices significantly influence microbial and metabolite composition of wine, altering the balance of sugars, organic acids, phenolic compounds, and volatile aroma constituents [67,68]. Furthermore, the wine microbial profile can be used to predict its metabolite profile. For example, the use of *Pichia guilliermondii* yeast in Chardonnays leads to the presence of the acid, ester, and lactone forms of a metabolite with C_6_H_10_O_2_ composition [65]. It has been demonstrated that fermentation dynamics—driven by yeast and bacterial strain selection, nutrient availability, and fermentation temperature—also modulate metabolic pathways involved in the fermentation process, leading to distinct profiles of amino acids, esters, and polyphenols. Similarly, postfermentation aging and storage conditions further influence wine molecular composition and chemistry, with time-dependent oxidation, polymerization, and hydrolysis reactions impacting the stability and bioavailability of bioactive compounds [69]. For instance, a study investigating the effect of aging (24 mo) on polyphenol content in Syrah red wine showed a significant decline in native grape polyphenol levels (anthocyanins, flavanols) and an increase in pyranoanthocyanins, ethyl-linked pigments, and flavanol-sulfonate content throughout the aging process [70]. Metabolomics platforms have been instrumental in detecting and characterizing these variations in primary and secondary metabolite production, offering deeper insights into the intricate connections between environmental, microbial, and aging factors that define wine’s unique molecular fingerprint.

Wine’s metabolites not only influence the quality and organoleptic properties of wine but also contribute to its biological effects. In the last decade, a growing body of in vitro and in vivo studies supported by metabolomics data has furnished new evidence on the wide range of health-promoting activities of dietary and wine-derived polyphenols, including their anti-inflammatory, antioxidant, anticarcinogenic, anti-adipogenic, antidiabetic, and neuroprotective potentials [[71], [72], [73], [74], [75]]. Metabolomics is particularly helpful in nutritional intervention studies, enabling the assessment of metabolic responses to wine consumption, the identification of novel biomarkers of wine intake, and the characterization of individual metabolic phenotypes in response to dietary modulation [63,76].

In a crossover intervention study conducted by Haas et al. [48], the untargeted plasma metabolomic analysis from fasting blood samples showed significant changes in 39 metabolites after red wine consumption (250 mL/d, 5 d/wk) for 3 wk, particularly those associated with pathways involving amino acids, vitamins and cofactors, lipids, and carbohydrates. For instance, significant increases were observed in the concentrations of metabolites of the pentose phosphate, ascorbate, and aromatic amino acid pathways, including sedoheptulose, arabinose, ribitol, arabitol, xylitol, gulonate, and indole propionate. Similarly, concentrations of the glutamate metabolites citramalate and N-acetylglutamine, the branched-chain amino acid metabolites 3-methyl-2-oxovalerate and 2,3-dihydroxyisovalerate, as well as lanthionine (cysteine metabolism) and acisoga (polyamine metabolism), were elevated after red wine consumption.

In a recent systematic review, Lekka et al. [77] compiled 51 studies from the last 25 y, investigating shifts in metabolic profiles within blood, urine, and feces samples, encompassing both short-term and long-term studies of the consumption of red and/or white wine. Their assessment also included derivatives such as dealcoholized wine, red wine extracts, and grape juice. The authors identified ∼600 compounds showing statistically significant increases in one or more of the tested matrices when comparing pre- and post-consumption. After removal of duplicates and exclusion of unknown entities from nontargeted analyses, a refined list of 361 annotated compounds was categorized based on chemical structure, including 125 phenolic compounds, 84 lipids and lipid-like molecules, 40 organic acids, 38 amino acids and derivatives, 14 carbohydrates and carbohydrate conjugates, 45 other organic compounds, and 5 vitamins and energy-related metabolites including nicotinic acid, pantoic acid, and isocitric lactone [77]. Such analysis not only highlights the diverse array of metabolites influenced by wine consumption but also provides valuable insights into the complex interplay between gut microbiota and host metabolism, shedding light on their roles in human health [42,43]. The substantial number of unidentified compounds reported in metabolomics studies related to wine and wine consumption emphasizes the need for further research to uncover chemical structures and their potential bioactivities.

### Glycomics

Glycomics is a powerful analytical technology to characterize carbohydrates (sugars), including polysaccharides (PS) and OS present in food and wine [78]. Complex PS and OS in wine are functional compounds that affect wine quality, chemical properties, and sensory perception (for example, astringency), and these factors could potentially convey human health benefits as prebiotics [[79], [80], [81]]. The wide range of PS and OS present in wine is derived from the pulp, seeds, and skin of grape berries as well as from yeast and bacteria during the winemaking process. However, information about the composition and structure of wine OS is still very limited [81]. Advances in mass spectrometry platforms and workflows over the last 15 y are starting to reveal the complexity and diversity of OS and PS structures in red, white, and sparkling wines. Significant efforts have been made to fractionate, isolate, and characterize OS in red and white wines produced from different grape cultivars to determine monosaccharide composition, glycosidic linkages, degree of polymerization, and OS structure, as recently reviewed by Apolinar-Valente et al. [81]. The abundances and structures of OS in wine have been found to depend on the cultivar and origin of grapes, the maturity stage of cultivars at harvest, and winemaking techniques such as grape pressing cycle conditions, contact time between skin and must, and the addition of commercial enzymes. For instance, total OS concentrations in wine and sparkling wines have been shown to vary greatly from 79 mg/L to 550 mg/L. The OS content is reportedly highest in red Syrah and Carignan wines (480 mg/L and 330 mg/L, respectively), followed by Merlot and Monastrel wines (252 mg/L and 244 mg/L, respectively) from France and Spain, Grignolino wine (127 mg/L) from Italy, Chardonnay wine (102 mg/L) from California, and Tempranillo and Verdejo sparkling wines (109–112 mg/L and 79–84 mg/L, respectively) from Spain [[82], [83], [84], [85]]*.* The influences of terroir and vintage on wine OS concentration, composition, and structure were also demonstrated in a few wine cultivars (for example, Monastrell and Tempranillo Spanish red wines) [79,86].

Complex OS in foods such as human and cow milk and fruits are bioactive molecules that modulate the gut microbiota community and function. In this capacity, OS can exert antimicrobial properties against pathogenic bacteria by preventing their adhesion and invasion of intestinal epithelial cells [87]. Furthermore, OS such as arabinoxylan-OS can simultaneously display a prebiotic effect by promoting the growth of health-promoting gut microbes, including *Lactobacillus* as well as *Bacteroides–Prevotella* and *Clostridium coccoides*–*Eubacterium rectale* groups, which can impact human health [88]. Despite significant efforts dedicated to the discovery of bioactive OS in wine and grape byproduct (pomace) [89,90], more studies are required to fully characterize the chemical structure and composition of wine OS and potential links to gut function and health. Such efforts may also help guide the development of new functional PS and OS-focused food products or upcycled ingredients with desired health effects.

### Lipidomics

Historically, research on wine and lipids was limited to well-characterized classes of lipids, such as fatty acids, focusing on their profiles in the different stages of wine production. In recent years, advancements in analytical technologies—particularly advanced chromatographic techniques coupled with high-resolution mass spectrometry as well as development in sample preparation workflows—have revolutionized the study of wine lipids through the development of comprehensive targeted and untargeted lipidomics platforms. These approaches have facilitated the identification and quantification of a broad spectrum of lipid classes such as phospholipids, glycolipids, sterols, and sphingolipids, with emerging evidence highlighting their functional role in grapevine health, fermentation, and wine’s organoleptic properties [91]. For instance, lipids such as medium-chain fatty acids and unsaturated fatty acids, including linoleic acid and linolenic acid, can stimulate the formation of yeast volatile metabolites during fermentation and constitute a major source of wine aroma precursors, influencing the overall composition and sensory properties [92]. Moreover, lipidomic analysis applied to Pinot Noir wines from different growing areas revealed distinct lipid signatures that could be used as markers to predict and authenticate wine provenance [93]. Despite these significant advancements, a critical gap remains in our understanding of the potential health implications of wine-derived lipids. Certain lipid classes, such as PUFAs and phytosterols, are well known for their health-promoting properties, particularly within the Mediterranean diet, where they have been linked to increased longevity, improved gut health, and a reduced incidence of chronic diseases [[94], [95], [96]]. Although these lipids have been detected in wine [97], their bioavailability, metabolism, and physiological impact after wine consumption remain largely unexplored. Furthermore, the potential gut microbial generation of xenolipid derivatives using wine lipid substrates is completely uncharacterized. This presents an exciting opportunity for future research to bridge the fields of lipidomics, nutrition, and health, advancing our understanding of how the intricate lipid matrix of wine and diet may influence human physiology and gastrointestinal function.

### Proteomics and peptidomics

Proteomics studies that characterize the structure, function, and interactions of the proteins within biological systems have identified proteins in wine that originate from grapes, yeast, and other microorganisms during fermentation [98]. Although grapes exhibit significant protein diversity, only a fraction persists in the final wine product, with the majority being removed on bentonite treatment during clarification steps, or degraded into a variety of peptides throughout the vinification process [99]. Despite the low level of residual proteins in wine (ranging from 10 to 300 mg/L) due to denaturation and proteolysis of the grape and yeast proteins during fermentation, they remain an important factor influencing wine stability and quality [100]. Beyond their physicochemical contributions, emerging proteomic and peptidomic studies suggest that certain peptides derived from wine proteins may exhibit bioactive properties. For instance, peptides are released through yeast autolysis, a process that occurs during wine aging and is characterized by the enzymatic degradation of intracellular components after cell lysis [99,101]. Among these peptides, those exhibiting angiotensin-converting enzyme inhibitory activity, which may contribute to blood pressure regulation, and prolyl endopeptidase activity, which is implicated in gut health, have been detected in both red and white wines. The concentrations of peptides in wine fluctuate throughout the fermentation and aging processes; an initial increase is observed during alcoholic fermentation due to proteolytic cleavage of grape and yeast proteins, followed by partial peptide utilization by lactic acid bacteria during malolactic fermentation as a nitrogen source. Subsequent autolytic degradation of yeast cells during aging leads to further peptide release through the action of hydrolytic enzymes such as proteases and peptidases [102]. A recent study identified over 2600 yeast (*Saccharomyces cerevisiae*) peptide sequences released in synthetic grape must after fermentation on lees (sediment generated in the winemaking process, composed of expired yeast and other solid particles such as grape skins and salts) [101]. Notably, in silico analysis of the top 200 most abundant peptides predicted bioactive properties, including anticancer, antihypertensive, antimicrobial, and immuno- and cyto-modulatory activities [101,103]. The bioactive peptide composition of fermented foods has been extensively characterized and represents an active area of investigation in “foodomics” [[104], [105], [106], [107]]. Kimchi, sourdough, and kefir have been shown to contain peptides with diverse biological activities, including antioxidant, antihypertensive, antimicrobial, anti-inflammatory, antithrombotic, hypolipidemic, and hypocholesterolemic effects [104,105,[108], [109], [110], [111]]. Of particular interest are antimicrobial peptides, which contribute to gut microbiota modulation by inhibiting the proliferation of pathogenic microorganisms although supporting the growth of beneficial bacteria. Despite these interesting observations, the characterization of bioactive peptides in wine remains largely unexplored.

### Ionomics

Over the last decades, comprehensive profiling of multielement composition (termed “ionomics”) has significantly increased among viticulturists and enologists due to the need to determine wine toxicological profile, geographical origin, and quality. Analyses of trace elements in wine have been carried out by atomic absorption spectroscopy, inductively coupled plasma atomic emission spectrometry, or inductively coupled plasma mass spectrometry [112]. The elemental composition in wines is classified into macro elements such as Ca, K, Na, and Mg, present at levels between 10 and 1000 mg/L, followed by microelements, such as Al, Fe, Cu, Mn, Rb, Sr, and Zn, present in the range of 0.1–10 mg/L. Trace elements include Ba, Cd, Co, Cr, Li, Ni, Pb, and V that are present in the ppt range (0.1–1000 μg/L) [113].

Select elements such as Fe, Cu, K, Ca, Zn, and Al significantly influence wine’s overall quality and organoleptic characteristics such as aroma, taste, color, and health benefits [114,115]. Certain metals, such as Cu, Zn, and Mn, act as cofactors for microbial enzymes, influencing gut microbiota composition and metabolic pathways related to SCFA production, bile acid metabolism, and oxidative stress regulation [116]. Notably, Cu(II) chelation in red wine has been shown to increase the radical scavenging activity of gallic acid, catechin, and caffeic acid, suggesting a potential role in mitigating inflammation and oxidative stress—key factors in gut health [117]. Conversely, excessive intake of certain trace elements can lead to detrimental health effects, including gastrointestinal symptoms, muscle pain and have negative effects on gut microbial diversity [116,118]. Moreover, toxic elements like Pb and Cd can disrupt gut barrier integrity and microbiome balance, underscoring the importance of ionomics in contaminant detection and quality control [119]. Integrating ionomics to other multiomics technologies (for example, metabolomics, metagenomics, and proteomics) offers a powerful approach to consider potential functional synergies between minerals, phytochemicals, OS, peptides, lipids, and other metabolites derived from wine, and to better understand how wine’s elemental profile interacts with gut microbial ecosystems.

## The Wine-Food Matrix and Future Research Directions

Dietary patterns and specific foods will likely influence the interactions between wine components, the gut microbiota, bioavailability of wine bioactives, and host physiology. Put differently, wine’s effects on health likely involve complex interactions impacted by the broader dietary pattern and specific foods co-consumed with wine.

### Wine and the Mediterranean diet

The Mediterranean diet, which is rich in fiber, fruits, vegetables, and healthy fats, may contribute to complementary compounds that interact with wine-derived metabolites [120]. The matrix in which wine is consumed—that is, meal composition and food preparation methods—can significantly influence the release, absorption, bioavailability, and metabolic pathways of both wine and food bioactives [121]. Although these interactions are increasingly recognized for their influence on physiological processes, including postprandial oxidative stress, lipid metabolism, and glycemic response, their underlying mechanisms remain largely unexplored [122,123]. The health impacts of the Mediterranean diet in combination with moderate wine intake have been considered in several studies. Evidence suggests that this combination enhances cardiometabolic health, reduces the risk of noncommunicable diseases, and slows cognitive decline with aging [120,122,124,125]. For instance, a cross-sectional analysis of the Prevención con Dieta Mediterránea trial, which assessed the Mediterranean diet’s effects on CVD prevention in high-risk individuals, found that red wine consumption (≥1 drink/d) was associated with a lower prevalence of metabolic syndrome [126]. Similarly, adherence to the Mediterranean-Dietary Approaches to Stop Hypertension (DASH) Diet Intervention for Neurodegenerative Delay, a hybrid dietary approach that combines elements of the Mediterranean diet (including 1 glass of wine/d) and the DASH has been positively associated with cognitive health [127,128]. Wine is also widely consumed outside the well-studied Mediterranean diet framework. Thus, understanding how wine interacts within more diverse nutritional contexts is critical to accurately assess its health implications and develop context-appropriate dietary guidance.

### Food matrix and compound bioavailability

Relevant to potential food–wine interactions, a study using the Simgi gastrointestinal digestion system evaluated the interactions between lipids and red wine [123]. Lipids (9.9 g of olive oil and 343.8 mg cholesterol) and red wine (225 mL) codigestion tended to increase the percentage of bioaccessible monoglycerides and reduce cholesterol bioaccessibility from 80% to 49%, which is consistent with the effect of the grape seed polyphenols gallic acid, catechin, and epicatechin on the inhibition of pancreatic cholesterol esterase, bile acid-binding capacity, and the solubility of cholesterol micellization [129]. Interestingly, a colonic fermentation in vitro model resulted in significantly higher growth of lactic acid bacteria and *Bifidobacteria*, along with greater production of total SCFAs, in the red wine and lipids model compared with the control. Tamargo et al. investigated how food matrix interactions influence red wine-polyphenol bioaccessibility and bioactivity using the Simgi dynamic digestion model. The study found that codigestion of wine with glucose or whey proteins reduced in vitro glucose absorption by over 50% and slowed α-lactalbumin degradation, although significantly altering polyphenol profiles—specifically increasing by 70% the bioaccessible fraction of (−)-epicatechin. Additionally, these interactions modulated the microbiota community, reducing bacterial diversity although promoting “beneficial” taxa such as *Akkermansia* and *Bifidobacterium*. Notably, codigestion enhanced the production of butyric acid, a SCFA linked to various gut health benefits [130]. Given the influence of the wine-food matrix on the bioaccessibility and composition of wine-derived metabolites, and the wine-associated microbial composition shifts in the artificial gut models, future research exploring how these factors affect host physiology across diverse dietary patterns and foods in vivo is warranted.

Understanding how food impacts the bioavailability of wine-derived compounds is critical to characterize their physiological effects in target tissues. Polyphenol bioavailability varies significantly among compounds, in most part due to their structural diversity and biotransformation on absorption. Wine-derived polyphenols undergo phase I reactions, involving oxidation, reduction, and hydrolysis, followed by phase II conjugation reactions, including sulfation, glucuronidation, and methylation, whereas transport proteins facilitate their cellular distribution, metabolism, and excretion, collectively regulating their clearance, bioactivation, and biological effects [[131], [132], [133], [134], [135], [136], [137], [138]]. Additionally, deconjugating enzymes produced by gut microbiota can reverse these processes, regenerating bioactive forms of polyphenols and extending their biological activity [137]. Vitaglione et al. [139] investigated the bioavailability of *trans*-resveratrol in humans after wine consumption (300–600 mL/meal) in a broader dietary context consisting in 4 groups: fasting, standard meal (“Milanese beef cutlet”: beef, egg, bread-crumbs, fried in maize oil and chips), fat meal (1321 kcal and lipid content of 69 g) and lean meal (1200 kcal and lipid content of 3.3 g). Both fat and lean meals were set up with 3 courses containing pasta and peas as the first course, a slice of chicken meat and salad as the second course, and a cup of mixed fruits as the third course. The results indicate that although *trans*-resveratrol is rapidly absorbed, its bioavailability remains extremely low due to extensive metabolism into glucuronide derivatives, with meal composition having minimal influence. Although these findings provide insights into the pharmacokinetics of *trans*-resveratrol, the influences of disparate meals and foods on bioavailability of the hundreds to thousands of wine-associated metabolites in the gastrointestinal tract remains an open area of research.

A recent review highlights the importance of the wine alcohol content in enhancing the bioavailability of wine’s beneficial compounds [140]. Similar to how dietary sugar, cholesterol, and saturated fat exert different health effects depending on the food matrix, alcohol’s health impact likely depends on the matrix in which it is consumed. For instance, ethanol facilitates the solubility and absorption of certain phenolic compounds associated with cardioprotective properties, such as tyrosol, hydroxytyrosol, and resveratrol [141,142]. This could, at least partially, explain why nonalcoholic alternatives like grape juice represent a less effective matrix as compared with red wine in the absorption of the polyphenols quercetin and resveratrol [143]. Similarly, studies using red wine extract or dealcoholized red wine often fail to replicate the cardioprotective effect observed with unmodified red wine [[144], [145], [146], [147], [148]].

### Bioregional considerations to study wine-food–gut interactions

Most studies exploring the impact of ingested compounds on gut physiology or microbiota biology utilize biosamples like serum, urine, and feces, which capture important aspects of systemic and gut lumen metabolism but offer limited insight into the metabolic processes occurring throughout the entire digestive tract. Future studies should expand sampling to all regions of the gut to better understand how wine consumption (with or without specific foods or in the context of a specific dietary pattern) changes the local molecular milieu. New technologies have recently enabled sampling the content of small and large intestine regions, demonstrating the profound differences in the fasting and postprandial metabolome along the gastrointestinal tract [149,150]. Advanced gastrointestinal simulators could complement these approaches and allow for controlled manipulation of dietary components coupled to various wines differing in molecular compositions (see Food matrix and compound bioavailability section). Additionally, the extensive microbial transformation of wine-derived compounds in the gut presents a challenge in distinguishing metabolites directly originating from wine from those generated by the microbiota or microbiota–host cometabolism. This differentiation is critical for elucidating the absorption, distribution, metabolism, and excretion of specific wine metabolites in vivo and for assessing their bioactive properties and potential health implications. The use of human stool bacteria cultures (or cultures with microbes from other gastrointestinal regions) will prove useful to determine microbial xenometabolites produced in the presence of wine, food components, and wine components.

### Interindividual variability in responses to moderate wine consumption

Finally, person-to-person variability in terms of health-relevant responses to wine or wine and food remains an unmapped area of research. Differences in gut microbiota composition, genetics, enzymatic activity, (patho)physiological status, gastric and gastrointestinal motility, and lifestyle factors could lead to distinct metabolite profiles and variations in how individuals metabolize wine-derived compounds [151]. This variability extends to wine-food interactions, as similar dietary patterns may elicit distinct physiological responses to wine-derived compounds based on an individual’s unique microbiome and metabolic capacity [42,152]. Supporting this premise are results from research on dietary polyphenols, which have identified significant interindividual variability in metabolite excretion and production. Some individuals could be classified as high or low excretors of flavonoids, phenolic acids, prenylflavonoids, alkylresorcinols, and hydroxytyrosol, whereas others differed in their ability to produce bioactive metabolites such as urolithins from ellagitannins or equol from isoflavones [151]. A recent interventional study identified 3 distinct metabotypes, each exhibiting unique variations in microbial metabolism, metabolite production, and nutrient bioavailability in response to moderate wine consumption [153]. Genetic polymorphisms in metabolic enzymes involved in phase I and phase II reactions have been proposed to contribute to variability in the metabolic response to wine consumption [151]. Variants in cytochromes P450 genes have been shown to alter enzyme activity, thereby influencing the bioavailability and metabolic fate of polyphenols [138]. Similarly, polymorphisms in phase II enzymes have been associated with differences in the excretion and activity of phenolic metabolites, impacting their potential health effects [154,155]. These genetic differences not only influence wine-polyphenol pharmacokinetics but also modulate their gut microbiota interaction and downstream physiological effects, which could in theory lead to diverse health outcomes across individuals. To address person-to-person variability, future studies should focus on comprehensive physiological phenotyping and multiomics profiling of individuals after acute (postprandial) and longer-term consumption of wines and food.

In conclusion, although this narrative review highlights the potential gut-related benefits and effects of wine-derived polyphenols and other bioactives, these factors must be considered alongside the broader health implications of ethanol consumption. In alcohol-dependent individuals (118.9  g/d of alcohol for >10 y), there is evidence for gut dysbiosis [[156], [157], [158], [159], [160]]. Recent meta-analyses and public health reports suggest that even moderate alcohol intake may increase the risk of some cancers (for example, breast and colorectal), contribute to liver dysfunction, and compromise intestinal barrier integrity, potentially leading to systemic inflammation [2,[161], [162], [163]]. However, when considering the ethanol intake literature to help shape dietary guidelines and public health messaging, there is a significant interpretive caveat: the diverse forms and contexts in which ethanol is consumed will likely impact health outcomes differently. It is reasonable to consider, for instance, that moderate wine consumption coupled to nutrient-rich meals would convey a different health and physiology profile compared with regular intake of distilled spirits sans food.

The intricate relationships among wine, food, and gut health call for a transformative change in how we think about the potential health implications of moderate wine consumption. Traditional approaches, centered on isolated compounds such as resveratrol or ethanol, fail to capture the complexity of wine and its frequent consumption with food. To achieve a paradigm shift, a holistic approach is crucial—one that acknowledges the complex molecular composition of wine, the interplay of wine-associated factors with dietary patterns and specific food components, the influence of gut microbes on bioavailability and conversion of wine components, and interindividual differences in metabolic responses to moderate wine consumption. Multiomics technologies offer powerful tools to comprehensively profile wine's complex molecular composition and to explore its interactive effects with food components. Integrating metabolomics, glycomics, lipidomics, proteomics, and ionomics will help build detailed molecular maps of wine matrices, capturing both native compounds and their transformation products during digestion and microbial transformation. Coupled with bioactivity screening and clinical validation, these innovative approaches are expected to streamline the identification of specific wine-food pairings that amplify beneficial effects on gut microbiota and overall health.

## Author contributions

The authors’ responsibilities were as follows – AA, MB, KH, HJ, HHS, SHA, JBS: contributed to the conception, design, writing and final content of the manuscript; and all authors: read and approved the final version of the manuscript.

## Funding

This work was supported by the University of California, Davis Innovation Institute for Food and Health.

## Conflict of interest

SHA reports a relationship with XenoMed LLC (dba XenoMet) that includes: equity or stocks. JBS reports a relationship with VinZymes, Incorporated that includes: equity or stocks. HHS reports a relationship with VinZymes, Incorporated that includes: equity or stocks. HHS reports a relationship with Sonomaceuticals, LLC that includes: consulting or advisory. SHA is an Associate Editor for *Advances in Nutrition*. If there are other authors, they declare that they have no known competing financial interests or personal relationships that could have appeared to influence the work reported in this paper.
