# Supplementary material for: A Multiomics Framework to Unlock the Relationships between Wine, Food, and Gut Health
Source: Adv Nutr. 2025 Jun 21;16(8):100468. doi: 10.1016/j.advnut.2025.100468 (PMC12296477; doi:10.1016/j.advnut.2025.100468)
Supplement: multimedia component 1 [file mmc1.docx]

***A multi-omics framework to unlock the relationships between wine, food, and gut health (Abrieux et al.)***

| **Body System** | **Cardiovascular System** | **Respiratory System** | **Digestive System** | **Nervous System** | **Endocrine System** | **Muscular System** | **Skeletal System** | **Immune System** | **Integumentary System** | **Excretory System** | **Reproductive System** |
| --- | --- | --- | --- | --- | --- | --- | --- | --- | --- | --- | --- |
| **Associated Search terms** | Cardiovascular | Respiratory | Gut health | Nervous system | Endocrine | Muscle | Bone | Immune | Skin | Urinary | Reproductive |
|  | Circulatory | Pulmonary | Microbiota | Brain | Hormones | Myocytes | Osteoblasts | Lymphatic | Cutaneous | Renal | Fertility |
|  | Blood Pressure | Lung | Dysbiosis | Cognition | Adrenal | Rheumatic | Osteoporosis | T cells | Dermatology | Kidney | Pregnancy |
|  | Heart | Asthma | Xenobiotic | Memory | Pancreas | Arthritis | Vitamin D | Lymphocyte | Cosmetic | Excretion | Estrogens |
|  | Hypertension | Erythrocyte | Gastrointestinal tract | Sensory | Thyroid | Skeletal muscle | Calcium Homeostasis | Cytokine | Collagen | Uric Acid | Genitalia |
|  | Atherosclerosis | Hypoxia | Inflammatory Bowel Disease | Glial | Melatonin | Proprioception | Rheumatoid Arthritis | Macrophages | Dermatitis | Prostate | Infertility |
|  | Vascular | Red Blood Cell | Liver | Neurodegenerative | Insulin | Exercise | Dental | Inflammation | Dermis | Bladder | Sexual Function |
| **Total Non-Redundant Papers Found** | 4114 | 765 | 1925 | 3406 | 1081 | 535 | 888 | 2579 | 2770 | 844 | 1239 |

**Supplemental Table S1. Publication numbers on Pudmed for the search terms “wine AND” each of the 11 body systems (e.g., cardiovascular, respiratory, digestive, nervous, endocrine, muscular, skeletal, immune, integumentary, excretory, reproductive) used to build the Treemap in Figure 1 of the paper**.

**Search strategy and selection criteria for the literature search reflecting the number of publications used to build the Treemap figure**.

To identify publications numbers for the search terms “wine AND” each of the 11 body systems used to build the Treemap in Figure 1 of the paper, the authors searched PubMed using the search strings “wine” [AND] a total of 7 associated search terms per body system. Duplicates within each body system list were removed, to obtain the total number of non-redundant publications. To find the number of publications specifically examining the link between wine and microbiota within the digestive system list, the authors manually screened for any papers with "microbiota," "microbiome," or "microbial" in the title and abstract.
